# Secondary Patellar Resurfacing in TKA: A Combined Analysis of Registry Data and Biomechanical Testing

**DOI:** 10.3390/jcm10061227

**Published:** 2021-03-16

**Authors:** Leandra Bauer, Matthias Woiczinski, Christoph Thorwächter, Oliver Melsheimer, Patrick Weber, Thomas M. Grupp, Volkmar Jansson, Arnd Steinbrück

**Affiliations:** 1 Department of Orthopaedics, Physical Medicine and Rehabilitation, University Hospital, LMU Munich, Marchioninistraße 15, 81377 Munich, Germany; matthias.woiczinski@med.uni-muenchen.de (M.W.); christoph.thorwaechter@med.uni-muenchen.de (C.T.); thomas.grupp@aesculap.de (T.M.G.); volkmar.jansson@med.uni-muenchen.de (V.J.); arnd.steinbrueck@med.uni-muenchen.de (A.S.); 2 German Arthroplasty Registry (EPRD Deutsche Endoprothesenregister gGmbH), Straße des 17. Juni 106-108, 10623 Berlin, Germany; melsheimer@eprd.de; 3 ECOM-Excellent Center of Medicine, Arabellastraße 17, 81925 Munich, Germany; patrick.weber@atos.de; 4 ATOS Clinic Munich, Effnerstr. 38, 81925 Munich, Germany; 5 Aesculap AG, Research and Development, Am Aesculap Platz, 78532 Tuttlingen, Germany

**Keywords:** TKA, secondary patellar resurfacing, registry data, kinematic, knee rig

## Abstract

The German Arthroplasty registry (EPRD) has shown that different prosthesis systems have different rates of secondary patellar resurfacing: four years after implantation, the posterior-stabilized (PS) Vega prosthesis has a 3.2% risk of secondary patellar resurfacing compared to the cruciate-retaining (CR) Columbus prosthesis at 1.0% (both Aesculap AG, Tuttlingen, Germany). We hypothesized that PS implants have increased retropatellar pressure and a decreased retropatellar contact area compared to a CR design, which may lead to an increased likelihood of secondary patellar resurfacing. Eight fresh frozen specimens (cohort 1) were tested with an established knee rig. In addition, a possible influence of the registry-based patient collective (cohort 2) was investigated. No significant differences were found in patient data–cohort 2-(sex, age). A generally lower number of PS system cases is noteworthy. No significant increased patella pressure could be detected with the PS design, but a lower contact area was observed (cohort 1). Lower quadriceps force (100°–130° flexion), increased anterior movement of the tibia (rollback), greater external tilt of the patella, and increasing facet pressure in the Vega PS design indicate a multifactorial cause for a higher rate of secondary resurfacing which was found in the EPRD patient cohort and might be related to the PS’ principle function.

## 1. Introduction

According to the German Arthroplasty registry (EPRD), 124,677 total knee arthroplasties (TKA) were performed in Germany in 2019 [1]. The main reason for implantation is degenerative joint wear. Due to demographic changes, it can be assumed that the number of artificial joints required by the population will continue to increase. Although total knee arthroplasty has developed rapidly in recent decades, up to 19% of patients are dissatisfied with their prosthesis [2,3,4,5].

There is controversy as to whether the patella should be replaced with a patella button in primary TKA [6,7,8,9,10]. In the United States (US), >90% of primary TKA are performed with patellar resurfacing [6,11]. In contrast, the Swedish Registry reports a significantly lower number (2.9%) of patellar resurfacing for primary TKA [12]. According to the EPRD, 88.9% of primary TKA in Germany are implanted without patellar resurfacing [1]. Since a patella button is rarely implanted as a primary implant in Germany, it is often necessary to implant a secondary patellar resurfacing if, e.g., anterior knee pain persists postoperatively. Out of 14,462 revisions in 2019 in Germany, 14.4% involved secondary patellar resurfacing [1]. The incidence of anterior knee pain after TKA is reported to vary in the literature, with values as high as 30% [13,14,15].

The German Arthroplasty registry lists individual prosthesis systems with corresponding implantation numbers, as well as the revision rate. It is noticeable that different prosthesis systems have different rates of secondary patellar resurfacing; for example, four years after implantation, the posterior-stabilized (PS) Vega prosthesis (Aesclap AG, Tuttlingen, Germany) has a 3.2% risk of secondary patellar resurfacing compared to the cruciate-retaining (CR) Columbus prosthesis (Aesculap AG, Tuttlingen, Germany) at 1.0%. These different rates might be related to femorotibial design parameters, such as PS or CR. The more restricted movement of PS systems may be related to this difference, but a biomechanical explanation has not been described in the literature. It has been shown that a PS design, compared to a medially stabilized design, results in higher retropatellar facet pressure [16]. This might also be transferred to the comparison of PS and CR.

In our study, we hypothesized that PS implants have increased retropatellar pressure and a decreased retropatellar contact area compared to a CR design. This could be identified as a cause of increased anterior knee pain within PS inlays, explaining the increased likelihood of secondary patellar resurfacing found in the registry cohort of the EPRD.

In order to test this hypothesis, the two above-mentioned prosthesis systems from the same manufacturer—with PS and CR systems with similar trochlea groove designs —were tested in an experimental setup. To simulate the in vitro situation, human specimens were used, representing cohort 1. In addition, a possible influence of the registry-based patient collective—cohort 2—was investigated.

## 2. Materials and Methods

### 2.1. EPRD Data

In 2012, the EPRD began recording arthroplasty care of hip and knee joints. This marked the beginning of the regular publication of annual reports on endoprosthetic procedures performed, facilitating a basis for evaluating hip and knee replacement. In recent years, revision rates for each knee implant system have also been listed. In addition, in the 2020 annual report, secondary patellar resurfacing numbers were included for the first time. An important indicator in the evaluation of knee prostheses is the probability of revision of the respective system. In order to indicate the probability of additional resurfacing of the patella, only fittings that had not already been treated with patellar resurfacing were considered. Secondary patellar resurfacing is considered a complementary operation in the EPRD that does not end the service life of the system. The amount of documented data is continuing to increase. In the current annual report, about 70% of the estimated total endoprosthetic procedures on knee and hip joints are covered. Participation in the collection of data from patients, hospitals, and statutory health insurers is on a voluntary basis. Follow-up of patients who may have been treated in a hospital without data capture for the EPRD is facilitated by hospitals providing mandatory billing data [1,17]. Data linkage creates a closed system, e.g., for tracking the survival analysis of the registry cohort.

This study, as part of EPRD data collection, was approved by the ethics committee of the Medical School of Kiel University (Approval number D 473/11).

### 2.2. Specimens and Implantation

Eight fresh frozen specimens were used for the experiments (cohort 1). Severe bone deformities or a varus/valgus ≥10° were exclusion criteria. These were checked by X-ray in two planes. The specimens were shortened 20 cm proximal and 15 cm distal to the joint line. In addition, unnecessary tissue was removed, and necessary tissue, such as capsules, ligaments, and tendons, was preserved. The relevant tendons were sutured into finger traps (Bühler-Instrumente Medizintechnik GmbH, Tuttlingen, Germany) with non-absorbable suture material (FibreWire, Arthrex, Munich, Germany) to simulate muscle forces on the knee rig. After the fibula head was fixed in the proximal tibia with a screw, the femur and tibia were embedded in metal pots with epoxy resin (Rencast FC53, Huntsman, Basel, Switzerland). After completion of the preparations, the Columbus prosthesis (Aesculap AG, Tuttlingen, Germany) fixed bearing with a CR inlay was implanted in the knee. Implantation was performed according to the tibia first technique. Standardized implantation was performed as in previously published studies [18,19,20,21]. The approach was subvastal to protect the quadriceps tendons of the joint for the use in the knee rig. Tibial and femoral incisions were performed with an intramedullary alignment. The rotation of the tibial components was aligned toward the medial third of the tibial tuberosity, and the femoral components were aligned along the trans-epicondylar axis. The femoral component was positioned centrally on the femur. This was followed by insertion of an inlay with a thickness of 10 mm in all specimens. Correct implantation was verified with a subsequent X-ray in two planes. After implantation and testing of the Columbus CR prosthesis, the Vega prosthesis was implanted with a PS inlay (both Aesculap AG, Tuttlingen, Germany) in the same specimen. The implantation was performed by the senior author, who is a highly experienced knee arthroplasty surgeon.

### 2.3. Biomechanical Setup

Biomechanical testing was performed on an established knee rig, which provides six degrees of freedom [18,19,21,22,23]. The knee rig performs knee flexion from 30° to 130° at a speed of 3°/s. A constant ground reaction force of 50 *N* is applied. The movement is actively controlled by the quadriceps force, while the muscle forces of the vastus medialis, vastus lateralis, semitendinosus, and biceps femoris muscles are simulated with 2 kg weights attached to the tendons. The quadriceps force was measured via a sensor on the tendon (8417-6002 Burster, Gernsbach, Germany). The actual flexion angle was calculated using data from two sensors attached to the hip and ankle (8820 Burster, Gernsbach, Germany). The test was controlled via LabView in real-time (Version 8.6, National Instruments, Austin, TX, USA). Measurement of the femorotibial kinematics was performed using an ultrasound motion analysis system (Zebris CMS 20, Isny, Germany). The required markers were attached to the femur and tibia. The first tests were performed to represent the native situation. Subsequently, the capsule was opened to sew in a pressure measurement foil behind the patella (K-Scan 4000, Tekscan, Inc., Boston, MA, USA). To prevent shear forces, a 0.1-mm-thick Teflon tape (PTFE tape) was glued to the sensor prior to the test.

### 2.4. Data Analysis

All further data processing was performed with MATLAB (MathWorks Inc., Natick, MA, USA). The pressure data were synchronized and interpolated with the flexion angles from the knee rig. Peak pressure (PP) was calculated by averaging the maximum value over a window with the eight surrounding values to avoid artifacts (according to [24]). The contact area (CA) reflected the number of pixels that exceeded a value of 0 MPa. Foil orientation was determined by tracing the ridge of the patella before each trial. Using this line, all print images were homogeneously aligned. Standardized orientation of the images allowed values for specific flexion angles to be averaged. The kinematics (tilt, shift, and spin of the patella; tibia rotation; anterior–posterior movement of tibia) were calculated using 3D motion data from an ultrasound device, employing an established method [25,26].

## 3. Results

### 3.1. EPRD Data

Table 1 lists the average age, gender distribution, and revision rate for the Columbus CR and Vega PS prostheses. On average, patients with a Columbus CR prosthesis were two years older (69 vs. 71 years). Gender distribution did not differ between the two designs (both 32% female, 68% male). The total number of implantations for each system shows that the Columbus CR system was implanted about 10 times more frequently as the Vega PS system. The probability of revision after one year was almost twice as high for the Vega PS than for the Columbus CR system, but in a good range of other PS TKA designs in the registry [1]. There was a similar trend for the probability of secondary patellar resurfacing after 1, 2, 3, and 4 years; this increased with time—the differences were not as clear at the beginning (0.3% vs. 0.2%) compared to after a period of four years (3.2% vs. 1.0%).

### 3.2. Pressure Data from Biomechanical Testing

The eight specimens (three females, five males), representing cohort 1, were on average 52 (±17.5) years old. The mean weight was 80.9 (±14.5) kg at a height of 174.2 (±7.9) cm.

**Figure 1:**
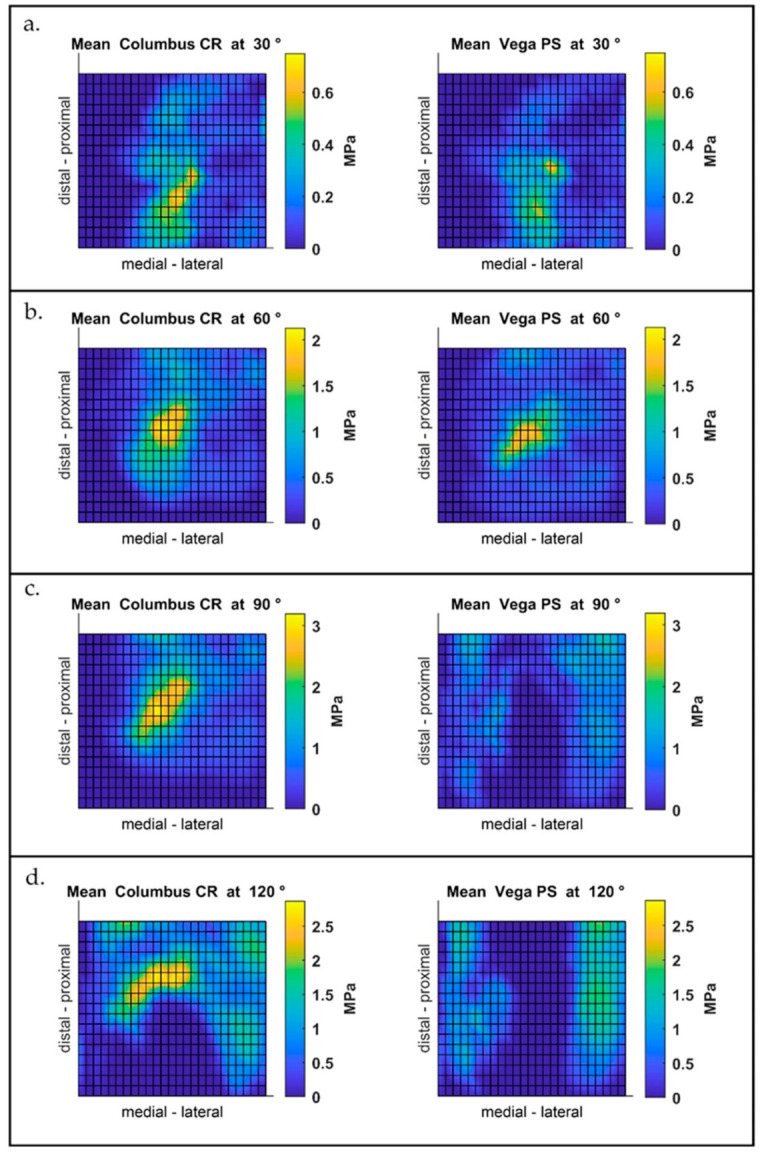
Mean pressure profiles of all specimens (*n* = 8) for four flexion angles ((**a**) 30°, (**b**) 60°, (**c**). 90°, (**d**). 120°). Left: Columbus cruciate retaining (CR). Right: Vega posterior stabilized (PS).

The mean pressure profiles from the Vega PS and Columbus CR prostheses are shown in Figure 1a–d. No significant differences between the prostheses could be observed at flexion angles of 30° and 60°. At a flexion of 90°, the distribution of surface pressure on the condyles could be observed with the Vega PS prosthesis, with more pressure on the patella facets. The increasing distribution on the patella facets continued up to a flexion angle of 120°.

**Figure 2:**
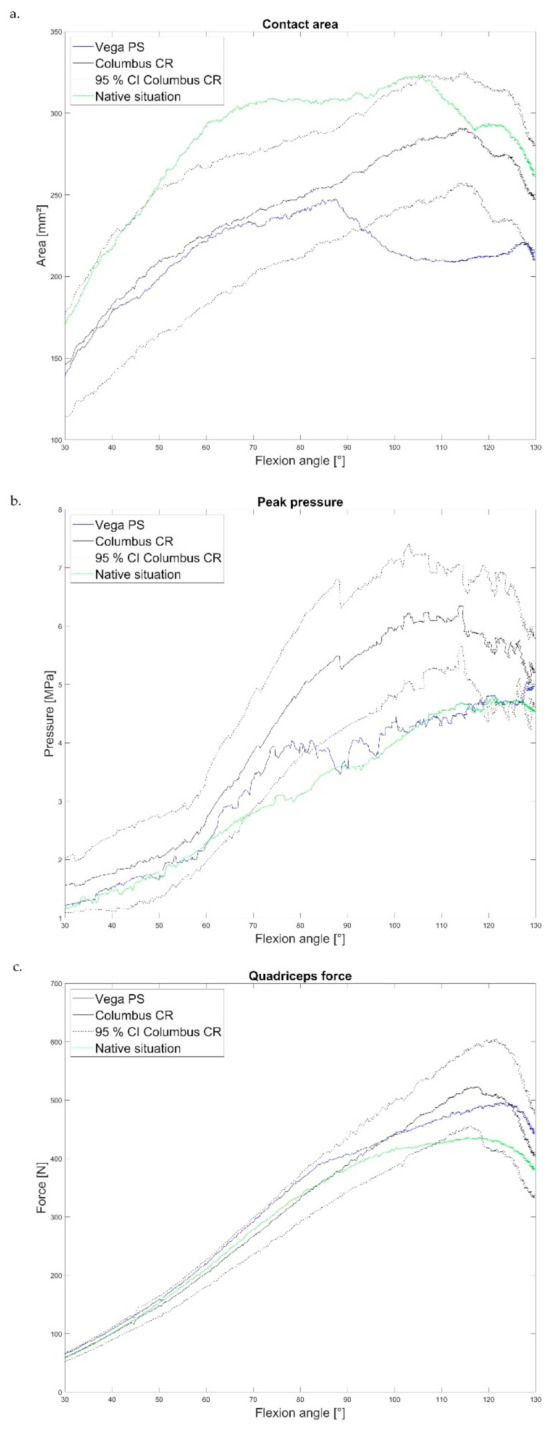
Mean values of all specimens (*n* = 8) for (**a**) retropatellar contact area, (**b**) retropatellar peak pressure, (**c**) quadriceps force of Vega PS (blue), Columbus CR (black), 95% CI of Columbus CR (black dashed), and native situation (green).

Figure 2a shows the mean values of all specimens of the CA. No significant differences in CA between Vega PS and Columbus CR were seen up to 90°. The CA of the Vega PS was always marginally lower. From 90°, the CA of Vega PS decreased significantly, while the CA of Columbus CR continued to increase with increasing flexion angle (until full range of motion, 130°). From 95°, the CA of Vega PS fell below the lower limit of the 95% confidence interval (CI). Thus, a significant difference was noted. The local minimum was reached at 200 N at 110° before the CA of Vega PS increased again slightly toward the maximum of the flexion angle.

The PP profile (mean values of all specimens) can be seen in Figure 2b. Here, a similar increasing PP course was observed up to 75° from the Vega PS and Columbus CR prostheses. Above 80°, the PP of Vega PS showed less of an increase compared to the PP of Columbus CR. The values fell below the lower limit of the 95% CI of Columbus CR—between 80° and 120°—showing a significant difference. The PP of Columbus CR increased steadily to a maximum of 6 MPa at 100° before the PP decreased slightly.

The quadriceps force (QF) is shown in Figure 2c. Up to 80°, the QF of Vega PS had higher values than Columbus CR. From this point, the QF showed a flattening of the curve for the PS design, while the QF of the CR inlay continued to increase to a maximum of 523 N.

### 3.3. Femorotibial and Patellofemoral Kinematics

**Figure 3:**
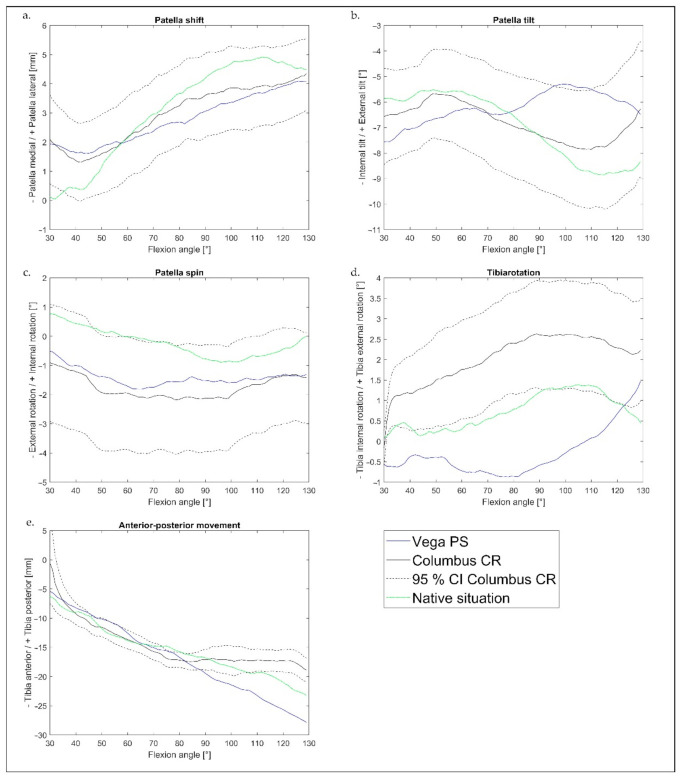
Mean kinematics of all specimens (*n* = 8) for the knee flexion angle (30°−130°): (**a**) patella shift, (**b**) patella tilt, (**c**) patella spin, (**d**) tibia rotation, and (**e**) anterior–posterior movement of tibia of Vega PS (blue), Columbus CR (black), 95% CI of Columbus CR (black dashed), and native situation (green).

Figure 3 shows the course of the mean values of all eight specimens of the kinematic—the patella shift, tilt, and spin over the flexion angle (30–130°). The Vega PS graphs were considered in relation to the mean curve of the Columbus CR prosthesis and its 95% CI. There was medial shift with increasing flexion in both systems. A greater external tilt could be detected in the PS system, increasing at a 75° flexion angle. The tilt of the Columbus CR was more similar to the native state. The spin motion showed no significant differences between the Vega and Columbus prostheses.

The femorotibial kinematics showed an increased anterior movement of the tibia (rollback) with the Vega PS design (Figure 3e). While the rollback of Columbus CR stagnated from 80°, it continued to increase for Vega PS. Vega PS showed increased internal tibial rotation compared to Columbus CR (Figure 3d).

## 4. Discussion

By analyzing the EPRD data of Columbus CR and Vega PS (cohort 2) in combination with biomechanical testing of eight specimens (cohort 1), a cause for the increased patellar resurfacing rates of Vega PS should be determined. Due to increased retropatellar pressure, we hypothesized that increased secondary patellar resurfacing had to be applied in patients with a Vega PS prosthesis.

The probability of revision for Vega PS and Columbus CR (cohort 2) increases annually. It should be determined in due course whether persistent pain is actually due to the anatomical conditions of the patella. Subsequently, there may be increasing degeneration of the retropatellar surface, which then requires resurfacing. At the registry-based patient level, no direct significant difference was found between Columbus CR and Vega PS patients (cohort 2). Data from the EPRD showed no significant age difference between the patient collectives of the two designs.

The most important finding in the biomechanical analysis (cohort 1) was that no significantly increased retropatellar pressure was observed with the Vega PS system. However, no increased CA could be detected either, which would explain the lower PP with Vega PS. From 100° flexion, the PS system requires less quadriceps force than CR to keep the ground reaction force constant. Increased anterior movement from 80° in the Vega PS design is due to the post cam mechanism. There is a possible forced movement in the knee joint caused by the implant. The external tilt of the Vega PS creates an altered pressure point with more strain on the patella facets compared to the native state. This could cause increased pain in patients.

Becher et al. compared a similar PS and CR system in a knee rig performing an extension movement. They also showed a reduced retropatellar pressure with the PS system compared to the CR system, which is consistent with our results [27].

Looking at the general data for CR and PS systems, it is striking that a CR design is implanted to such a large extent in Germany, with 42.9% CR vs. 19.0% PS designs [1]. In comparison, the PS design is most commonly implanted in the US, i.e., 45% [11]. Thus, the lower case numbers of PS designs also play a role in revision rates, as clinical experience has a significant impact on outcome [1]. This may also explain why PS systems perform worse on average than their CR counterparts. For a standard TKA patient, a PS system seems to be rarely used in Germany. If the posterior cruciate ligament is affected during surgery, a PS design may be implanted instead of a planned CR inlay. In addition, it cannot be directly determined whether patients with an implanted PS design had more severe preoperative varus–valgus deformities of the knee, which may lead to a worse outcome and thus an increased rate of secondary patellar resurfacing. A comparison with the literature shows that PS systems often performed worse compared to CR designs. In a retrospective study, Maney et al. investigated factors based on New Zealand Joint registry data that suggest a higher likelihood of secondary patellar resurfacing. They found an odds ratio for PS designs of 1.86 compared with CR designs in patients who had secondary patellar resurfacing. Therefore, a PS design is increasingly associated with secondary patellar resurfacing [28]. The increased rates of secondary patellar resurfacing with Vega PS compared to other PS systems could also result from the different forced kinematics. The Vega PS system actively intervenes in the kinematics from about 45° with an increased rollback. According to Arnout et al., other systems, such as the Nexgen LPS flex (Zimmer, Warsaw, IN, USA), force an increased movement beginning only from 102°, which means the post-cam mechanism is different to most other PS designs [29]. Fitzpatrick et al. demonstrate that post-cam geometry plays an important role in minimizing engagement velocity [30]. Thus, kinematics is influenced by the design of a PS system, which can be considered as a reason for increased secondary patellar resurfacing. The Vega system represents a PS design with more guided rollback, starting at an early flexion. Furthermore, the non-standard cam design may lead to internal rotation. Both of these could also affect our results.

There are also different rates for secondary patellar resurfacing within one design. Werth et al. evaluated 784 patients with TKA in a retrospective study. Five different implants with CR design were used. Different probabilities for secondary patellar resurfacing were found in the different implants: 0.8%, 1.4%, 1.6%, 6.2%, and 7.0%. Striking were the increased revision rates for two systems (7.0% for PFC Sigma and 6.2% for LCS, both DepuySynthes, Oberdorf, Switzerland). They noticed that the design characteristics of these two implants showed thicker trochlea design values than the others [31]. Biomechanical comparison of the trochlea thickness of Vega PS and Columbus CR, which was employed in our study, showed no difference in trochlea groove thickness and only a slightly greater external tilt of the trochlea in the Vega PS design.

The present study has some limitations. The results are limited by a low follow-up for revision rates. Revision rates are currently given for four years. It is possible that the figures could increase even more over a longer follow-up period or stagnate above a certain level. In addition, complete coverage of the register data is not yet provided. However, relatively reliable tracking of revisions can be assumed. Registry data do not include patient-reported outcome measurements (PROMS) or clinical examinations regarding anterior knee pain or existing instabilities. Since the patient-specific data are based on coded register data, no statements could be made about exact pre- or intraoperative conditions that could influence the results. Thus, conclusions concerning the patient cohort from the registry data are limited.

The biomechanical study was performed in vitro. This is necessary to study biomechanical parameters but does not replicate the direct in vivo situation. The in vitro study was performed on eight specimens, limiting any inference to the population. However, in vitro studies typically use around eight specimens [32]. The knee rig is used to perform a squat movement, but not an everyday movement such as walking or climbing stairs. Although parts of the performed movement can be transferred to everyday situations, they do not replace the actual movement of a TKA patient in everyday life. The implantation of the prosthesis has a strong influence on the outcome. Keshmiri et al. found a significant influence of the sagittal alignment component on the shift in an in vitro study on ten specimens [33]. Furthermore, the influence of the mediolateral component (lateralization, and medialization) on shift, tilt, and femoral roll-back was shown [32]. Posterior tibial slopes also influence kinematics, anterior–posterior movement, and tibial rotation in CR and PS designs [34]. However, both knee systems were implanted in the same specimen with the same bone cuts. The positioning of the implants of the knee systems should at least be comparable with the same specimen. Additionally, the processing of the data can influence the results—synchronizing the data afterwards can lead to errors in temporal accuracy. When recording 3D motion data, there may be gaps or jumps in the data; if this was the case, spikes were eliminated by linearization. In addition, the type of data analysis influences the result. Interpolation errors can never be completely excluded, but they are minimized.

## 5. Conclusions

In conclusion, no significant increased patella pressure could be detected with the PS design, but a lower contact area was observed. Lower quadriceps force (100°−130° flexion), increased anterior movement (rollback), greater external tilt of the patella, and increasing facet pressure in the Vega PS design indicated a multifactorial cause for a higher rate of secondary resurfacing, which was found in the EPRD patient cohort. The surgeon may want to consider performing primary patellar resurfacing when implanting a Vega PS prosthesis.

## Figures and Tables

**Table 1:** Information about Vega posterior-stabilized (PS) and Columbus cruciate-retaining (CR) prostheses from the German Arthroplasty registry (EPRD), representing cohort 2 [1].

	Vega PS	Columbus CR
Age (years)	69 (60–76)	71 (63–77)
Sex (f/m)	32/68	32/68
Total number of implantations	893	8648
Revision rate after 1 year (percent)	2.3 (600) ^1^	1.2 (6219) ^1^
Patellar resurfacing probability after 1 year (percent)	0.3 (557) ^1^	0.2 (5907) ^1^
After 2 years	1.6 (338) ^1^	0.7 (3826) ^1^
After 3 years	2.7 (199) ^1^	0.9 (2169) ^1^
After 4 years	3.2 (83) ^1^	1.0 (949) ^1^

^1^ Total numbers.

## Data Availability

Data are available on request due to ethical restrictions.
